# RAM, an RGDS Analog, Exerts Potent Anti-Melanoma Effects *In Vitro* and *In Vivo*

**DOI:** 10.1371/journal.pone.0025352

**Published:** 2011-10-03

**Authors:** Maria Simona Aguzzi, Daniela D'Arcangelo, Claudia Giampietri, Maurizio C. Capogrossi, Antonio Facchiano

**Affiliations:** 1 Laboratorio Patologia Vascolare, Istituto Dermopatico dell'Immacolata, IDI-IRCCS, Rome, Italy; 2 D.A.H.F.M.O. Section of Histology & Medical Embryology, Sapienza University of Rome, Rome, Italy; University of Bergen, Norway

## Abstract

Peptides containing the RGD sequence are under continuous investigation given their ability to control cell adhesion and apoptosis. Since small peptides are quickly metabolized and degraded *in vivo*, developing analogs resistant to serum-induced degradation is a challenging task. RGD analogs developed so far are known as molecules mostly inhibiting cell adhesion; this feature may reduce cell proliferation and tumor development but may not induce regression of tumors or metastases already formed. In the current study, carried out in melanoma *in vitro* and *in vivo* models, we show that RAM, an RGD-non-peptide Analog-Molecule, strongly inhibits cells adhesion onto plastic, vitronectin, fibronectin, laminin and von Willebrand Factor while it does not inhibit cell adhesion onto collagen IV, similarly to the RGDS template peptide. It also strongly inhibits *in vitro* cell proliferation, migration and DNA-synthesis, increases melanoma cells apoptosis and reduces survivin expression. All such effects were observed in collagen IV seeded cells, therefore are most likely independent from the anti adhesive properties. Further, RAM is more stable than the template RGDS; in fact it maintains its anti-proliferation and anti-adhesion effects after long serum exposure while RGDS almost completely loses its effects upon serum exposure. In a mouse metastatic melanoma *in vivo* model, increasing doses of RAM significantly reduce up to about 80% lung metastases development, while comparable doses of RGDS are less potent. In conclusion these data show that RAM is a potent inhibitor of melanoma growth *in vitro*, strongly reduces melanoma metastases development *in vivo* and represents a novel candidate for further *in vivo* investigations in the cancer treatment field.

## Introduction

Cell adhesion to cells and to the extracellular matrix controls different cellular functions, including survival, proliferation, migration and apoptosis [1], [2] and directly affects tissue plasticity and remodeling under both physiological and pathological conditions. The RGD (Arginine-Glycine-Aspartic acid) *motif* occurs in several extracellular matrix proteins; it is recognized by membrane-bound adhesion molecules and therefore plays a key role as cell adhesion mediator [2]. Peptides containing this *motif* show potent anti adhesion effects, since they compete for the integrin-matrix interaction and show anti-proliferative, anti-chemotactic and pro-apoptotic effects. Moreover, antibodies neutralizing α_v_β_3_ and α_5_β_1_ block integrin–mediated cell adhesion by antagonizing the RGD *motif* and completely inhibit tube formation in fibrin matrices [3]. Similarly, disintegrins molecules containing the RGD *motif* are known to block FGF-2-induced angiogenesis and B16F10 melanoma lung metastasis development in mice [4]. For the same reasons, molecules containing RGD motif immobilized onto appropriate matrices have pro-adhesive effects and RGD analogs may then elicit selective cellular responses such as wound healing, cell adhesion and migration [5], [6], [7]. Adhesive properties of RGD *motif* are been exploited for tumor imaging, targeting and radio treatment [8], [9], [10], [11], [12].

Small peptides have short circulation time since they are quickly proteolysed and metabolized *in vivo*. Peptides containing the RGD motif act via a competition mechanism [13], therefore high doses are usually required to reach *in vivo* effects, limiting the pharmacological use of RGD peptides and justifying the large interest to develop non-peptidic analogs with higher potency [14], [15], [16]. In order to overcome, at least in part, the issue regarding stability in serum, sequence modifications such as duplication and circularization [17], [18], synthesis of non peptidic analogs and liposomalization [19], [20], [21], [22] have been carried to enhance the stability and retention in the bloodstream.

We have previously demonstrated that RGDS peptide has additional effects not directly related to its anti adhesion activity. In fact we found that it is internalized into human endothelial cells and recognizes intracellular targets such as caspase-3, caspase-8 and caspase-9, leading to apoptosis most likely by an integrin-independent mechanism [23]. More recently we demonstrated that RGDS is internalized in melanoma cells, inhibiting their growth and inducing their apoptotic death with a mechanism independent of the extracellular anti adhesive activity [24]. According to studies published previously by us and by other Authors [23], [25], [26], such data indicate intracellular activity of RGDS-containing peptides highlighting novel pharmacological applications and suggesting novel intracellular targets.

We have previously shown that a novel RGDS analog named RAM (RGD-non-peptide Analog-Molecule) lacking peptidic bonds to overcome proteolytic degradation, mimicked the pro-apoptotic effects and the adhesive properties of RGDS on endothelial cells and showed potent anti angiogenesis activity *in vivo* [27]. Anti angiogenic and pro apoptotic molecules may have interesting applications in melanoma treatment and the previously demonstrated intracellular targets of RGDS opened novel fields of investigation; we then investigated RAM in a melanoma experimental setting and show in the present study that RAM exerts potent biological effects independently form the anti adhesive properties, is resistant to serum-induced loss-of-activity, and strongly inhibits progression of lung metastases in an *in vivo* melanoma mouse model.

## Results

### Effect of RGDS and RAM on SK-MEL-110 adhesion

**Figure 1:**
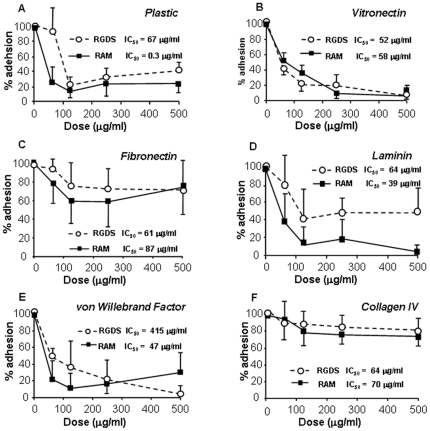
RGDS and RAM effect on melanoma cell adhesion. RGDS and RAM show relevant anti adhesive activity on cells seeded onto plastic (A), vitronectin 50 µg/ml (B), fibronectin 10 µg/ml (C), laminin 50 µg/ml (D) and von Willebrand Factor 50 µg/ml (E), while they do not show significant anti adhesive effects on collagen IV 50 µg/ml (F). The adhesion assay was carried out in the presence of 10% FCS and serial dilutions of RGDS or RAM. Cell adhesion was then quantified as optical density at 595 nm and was expressed as OD. These experiments were carried out three times in duplicate; mean ± S.D. is reported.

RAM was designed as a RGDS analog; its molecular structure is reported in Figure S1; in a previously published report we characterized RAM anti angiogenic activity *in vitro* and *in vivo* [27]. We and Others previously demonstrated that cell adhesion on collagen IV is RGD-independent [27], [28], [29]; as preliminary data necessary for the following experiments, we investigated the adhesion effects of RGDS onto SK-MEL-110 melanoma cells; Figure 1 shows that both RGDS and RAM inhibit adhesion of SK-MEL-110 seeded onto plastic, vitronectin, fibronectin, laminin and von Willebrand Factor in a dose-dependent way, with comparable potency and IC50 values, while they both lack relevant anti adhesion action onto collagen IV-seeded cells. We therefore concluded that RAM mimics RGDS anti adhesive as well as non-anti adhesive properties in melanoma cells. The adhesion-independent effects of RGDS and RAM were then investigated on collagen-IV seeded melanoma cells.

### RGDS and RAM effects on proliferation and migration of melanoma cells

**Figure 2 pone-0025352-g002:**
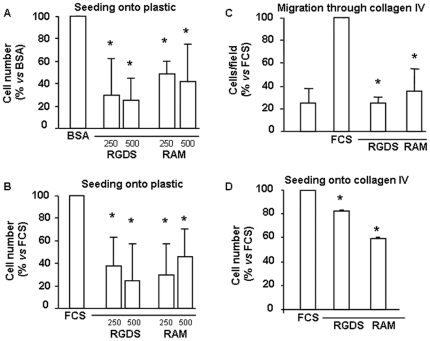
RGDS and RAM effect on proliferation and migration. RGDS and RAM show anti proliferation/anti migration effects onto plastic- and onto collagen IV seeded SK-MEL-110 cells. Proliferation of human melanoma cells seeded onto plastic was evaluated in the presence of RGDS and RAM (250–500 µg/ml) after 24 h treatment. RGDS and RAM significantly reduced melanoma cells proliferation either in the absence (A) and in the presence of serum (B) (* p<0.05) likely with an anti adhesive mechanism. (C) Serum-induced melanoma cells invasion through collagen IV (10 µg/ml) after 4 h incubation at 37°C was inhibited by RGDS and RAM (500 µg/ml) (* p<0.05) to a similar extent, likely with a non-anti adhesive mechanism. Forty-eight hours proliferation was evaluated onto collagen IV. RAM significantly inhibited cell growth under these conditions, while RGDS was less active. All proliferation and migration experiments were performed four times in duplicate and mean ± S.D. is reported.

**Figure 3 pone-0025352-g003:**
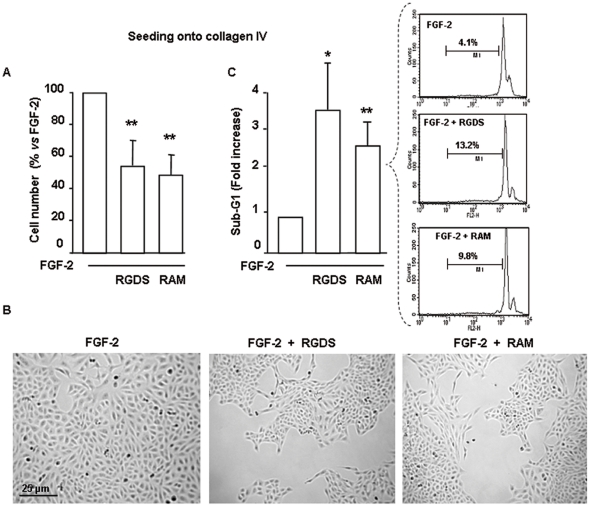
RGDS and RAM inhibit SK-MEL-110 proliferation and increase apoptosis with an adhesion-independent mechanism. (A) FGF-2 (10 ng/ml)-induced SK-MEL-110 proliferation on collagen IV (50 µg/ml) was evaluated after 48 h treatment in the presence of RGDS or RAM (500 µg/ml). Either molecules strongly inhibited cell proliferation (** p<0.01 vs FGF-2). (B) Representative images of experiments depicted in panel A are reported (scale bar = 25 µm). (C) SK-MEL-110 apoptosis was quantified as percentage of cells in sub-G1 phase, by FACS analysis of PI-stained cells after 48 h RGDS or RAM treatment (500 µg/ml) (* p<0.05 *vs* FGF-2 and ** p<0.01 vs FGF-2, respectively). Three independent experiments were performed and quantified and mean ± S.D. is reported; one representative experiment is shown (right side).

It is known that RGD peptides or RGD analogs exert anti proliferation and pro apoptotic effects by detaching cells from extracellular matrix, resulting in a caspase-dependent apoptosis (anoikis) [2], [19]. Under our experimental conditions, RGDS and RAM inhibited with similar potency proliferation of melanoma cells seeded onto plastic after 24 h incubation both in the absence and in the presence of serum (Figure 2A and B). Such effects most likely relate to the strong anti adhesive effect shown by RGDS and RAM onto plastic-seeded cells (Figure 1A). However, Figure 2C shows that both RGDS and RAM markedly reduced serum-induced melanoma cell migration through collagen IV, i.e. under experimental conditions where RGDS and RAM do not exert marked anti adhesive properties (see Figure 1F); under such conditions RGDS weakly but significantly inhibited FCS-induced proliferation of SK-MEL-110, while RAM showed strong and significant anti proliferation effects (Figure 2D); interestingly, RAM maintained a strong anti-proliferative effect on a different human melanoma cell line (SK-MEL-28), while the weak effect of RGDS was completely lost (Figure S2). Additional experiments indicated that RGDS and RAM significantly inhibit FGF-2-induced proliferation of collagen IV–seeded melanoma cells (46±16% and 53±14% inhibition, respectively, p<0.005) (Figure 3A), further demonstrating that RAM and RGDS show potent anti-mitogenic effect un-related to their anti adhesive action. Representative fields of such anti-proliferative action are reported in Figure 3B.

To analyze cell distribution in sub-G1-phase, melanoma cells were treated with RGDS or RAM for 48 h and stained with propidium iodide. Sub-G1 phase, considered as a marker of cell apoptosis, in the presence of FGF-2 was significantly increased (from 4% to 13.2% and to 9.8%) by RGDS or RAM treatment, respectively, in collagen IV -seeded cells, indicating pro-apoptotic properties most likely un-related to the anti adhesive action (Figure 3C). This pro-apoptotic effect was not present at earlier timepoint (i.e. 24 h incubation).

**Figure 4 pone-0025352-g004:**
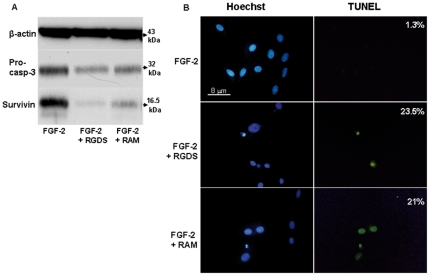
RGDS and RAM effect on apoptosis in human melanoma cells. (A) Apoptosis was confirmed by western blotting to detect pro-caspase 3 cleavage and survivin expression after RGDS and RAM treatment, in collagen IV seeded cells. (B) Apoptosis was also visualized by TUNEL staining and by nuclear fragmentation (Hoechst staining). Nuclei stained with Hoechst as well as TUNEL-positive nuclei were identified by means of a Zeiss Axioplan fluorescence microscope (original magnification, ×40).

Pro apoptotic effect in the presence of FGF-2 was confirmed by western blotting analysis investigating pro-caspase 3 cleavage. Both RGDS and RAM treatment reduced expression of caspase 3 inactive precursor (32 kDa) (Figure 4A). They also markedly reduced the expression of survivin, a member of the Inhibitor of Apoptosis Protein (IAP) selectively expressed during development and in proliferating cells and cancer cells [24].

The pro apoptotic effect was also quantified by TUNEL staining and was observed in cells treated with FGF-2 in the presence of RGDS or RAM (23.5% and 21.0% of total nuclei, respectively) while it was almost absent in FGF-2-only treated cells (1.3% of total nuclei) (Figure 4B).

All together figures 1 to [Fig pone-0025352-g002] [Fig pone-0025352-g003] 4 demonstrate with different approaches that RAM is a good functional analog of RGDS, and shows marked effects onto collagen-IV seeded melanoma cells, most likely unrelated to the anti adhesion activity.

### Stability test

**Figure 5 pone-0025352-g005:**
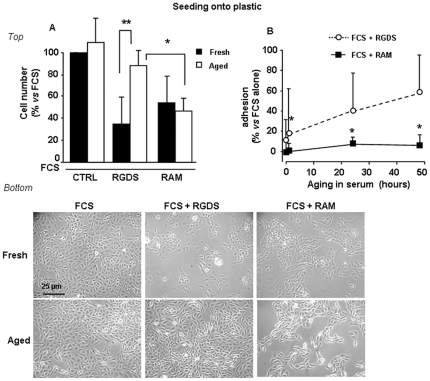
Stability test carried out in serum, in adhesion-dependent assays. (A) (*Top*) RGDS and RAM stability in serum was evaluated in a proliferation assay onto plastic-seeded cells. The molecules were either tested immediately after thawing (fresh molecules) or tested after 24 h pre-incubation at 37°C in 100% FCS (*aged* molecules). Under these conditions, RGDS (500 µg/ml) lost anti-proliferation activity compared to fresh RGDS (RGDS black *vs* RGDS white column), while RAM (500 µg/ml) maintained the ability to inhibit melanoma cells proliferation (RAM black *vs* RAM white column) (** p<0.01 *vs* FCS). In addition RAM *aged* molecule showed a significant higher anti-proliferation activity as compared to RGDS *aged* molecule (* p<0.05 RAM *vs* RGDS). Three independent experiments were performed and mean ± S.D. is reported. (*Bottom*) Representative images of treated cells are reported (scale bar = 25 µm). (B) RGDS and RAM were pre-incubated in 100% FCS at 37°C for increasing time points (0.5-1-24-48 hours) and then used in adhesion assay. Under these conditions, RAM significantly does not change its anti adhesive effect, while RGDS anti adhesive action is largely lost at 24 h (* p<0.05 RAM *vs* FCS).

We have previously shown that upon 24 h incubation in 100% FCS, RGDS completely lacks anti adhesive effect onto HUVEC, while RAM maintains the anti adhesive action similar to the fresh RAM molecule [27], [30]. Hence, in the present study we investigated whether the observed anti proliferation activity of RAM and RGDS onto melanoma cells is affected by 24 h pre-incubation in 100% FCS. “*Aged*” RAM (i.e., RAM kept in FCS 100% for 24 h at 37°C) inhibited SK-MEL-110 cells proliferation seeded onto plastic as much as the fresh molecule, while “*aged*” RGDS (i.e. incubated at 37°C for the same time) totally lost the anti proliferation effect compared to fresh RGDS (Figure 5A top). Representative images of treated cells are reported in Figure 5A (bottom).

To further investigate stability in the presence of serum, RGDS and RAM were pre-incubated in 100% FCS for increasing time points (0.5 h, 1 h, 24 h and 48 h) at 37°C and were then used in adhesion assays. Figure 5B shows that “*aged*” RAM maintained the strong anti adhesive action at all time points, while “*aged*” RGDS lost large part of its anti adhesive effect after 24 h and 48 h incubation in serum.

**Figure 6:**
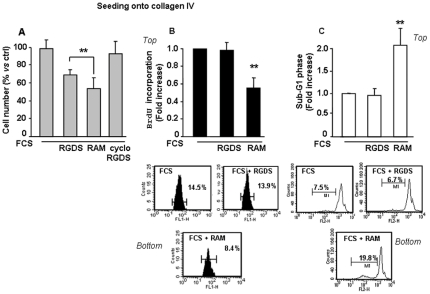
Anti proliferation effect on collagen-seeded cells upon serum induced degradation. (A) SK-MEL-110 cells were seeded onto collagen IV to allow RGDS- independent adhesion. RGDS, RAM and the control cyclo-RGDS were kept in 100% serum for 24 h in order to allow serum-induced degradation. Under such conditions RGDS and its analog cyclo-RGDS were inactive or significantly less active than RAM. (B) RGDS and RAM effect on DNA synthesis and apoptosis onto collagen seeded cells. RGDS and RAM (500 µg/ml) effect on DNA synthesis and apoptosis of melanoma cells was evaluated by FACS analysis. (a) (*Top*): DNA synthesis was analyzed by BrdU incorporation after 48 h treatment. While RAM in FCS reduced DNA synthesis, RGDS had no effect. (B*ottom*): Three independent experiments were performed and quantified; one representative experiment is shown (** p<0.01 vs FCS). (b) (*Top*): Apoptosis was quantified as percentage of sub G1-cell-phase by PI staining using FACS. RAM induced apoptosis under these experimental conditions, while RGDS had no pro-apoptotic effect (** p<0.01 vs FCS). Three independent experiments were performed and mean ± S.D. is reported. (B*ottom*): One representative experiment is shown.

To confirm that RAM is more potent and more stable than RGDS in the presence of serum-induced degradation, RGDS, RAM and the control cyclo-RGDS (a known RGDS analog with anti adhesion activity [31]), were kept 24 h in 100% serum, to allow serum-induced degradation. Proliferation was then measured in melanoma cells seeded onto collagen IV and treated with the RGDS or RAM or cyclo-RGDS. Such conditions investigated anti proliferation properties independent form the anti adhesive actions, upon serum-induced degradation. RAM strongly inhibited proliferation (about 50%), significantly more than RGDS (about 35%) (Figure 6A), while the control cyclo-RGDS, known anti adhesive analog of RGDS, was completely inactive, further suggesting that such anti proliferation effect is un-related to the anti adhesion action. RAM was also significantly more potent than RGDS either in inhibiting BrdU incorporation (Figure 6B) and by increasing sub-G1 phase (Figure 6C), indicating that RAM achieves its anti proliferation effect by increasing apoptosis and reducing DNA synthesis. Elucidating mechanisms underlying the weak residual anti proliferation effect of RGDS upon FCS-induced degradation, observed in Figures 2A, 5B and 6A requires further investigation and quantitative assays.

Other experiments indicate that RAM and RGDS action does not involve G2/M checkpoints regulation. In fact upon nocodazole synchronization, neither RAM nor RGDS affect cell cycle distribution.

All such data indicate that RAM is significantly more potent that RGDS in collagen IV– seeded melanoma cells, in experimental conditions mimicking the serum-dependent degradation, suggesting that RAM may represent a suitable candidate for further *in vivo* investigations.

### *In vivo* experiments

**Figure 7:**
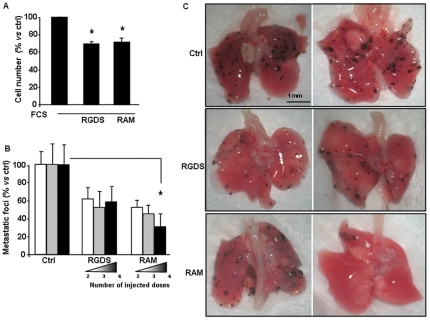
*In vitro* **proliferation of B16F10 and i** ***n vivo*** **experiments.** (A) In vitro proliferation of mouse melanoma cell line (B16F10) seeded onto collagen IV in the presence of serum, was significantly inhibited by RGDS or RAM (500 µg/ml). (* p<0.01 vs FCS). (B) RGDS and RAM were tested *in vivo* in a mouse melanoma lung-metastasis model. C57BL6/J mice were treated with B16F10 cells inoculated intravenously to induce lung metastasis formation. RGDS and RAM (2.6 mM) treatments were carried out according to three different injection schedules: i) at days 9^th^ and 11^th^ (2 total injected doses), ii) at days 7^th^, 9^th^ and 11^th^ (3 total injected doses) and iii) at days 5^th^, 7^th^, 9^th^ 11^th^ (4 total injected doses. At day 14^th^ mice were sacrificed, lungs were isolated and superficial macroscopic lung-metastases were counted. The number of metastatic foci in control mice was 124±40 (set to 100%). RAM significantly reduced the number of superficial metastases in a dose-dependent way (* p<0.05 *vs* control, by ANOVA), while RGDS was less effective. C) Representative lung-images are reported.

To investigate *in vivo* effects in a mouse model, we first tested *in vitro* the RGDS and RAM (500 µg/ml) effects on FCS-induced proliferation in a mouse melanoma cell line (B16F10) seeded on collagen IV. Both RGDS and RAM significantly reduced mouse melanoma cell proliferation by about 40% (Figure 7A). RGDS and RAM were then tested *in vivo* at the same molar dose (2.6 mM) in a mouse melanoma-lung metastasis model, according to procedures and doses previously identified for other RGD analogs [32]. B16F10 cells were injected at day 0 intravenously in C57BL6/J male mice (12 mice per group) to induce lung metastasis formation. Then RGDS and RAM treatments were injected into vein tail according to three different treatment schedules: i) at days 9^th^ and 11^th^ (i.e., 2 total injected doses), ii) at days 7^th^, 9^th^ and 11^th^ (i.e., 3 total injected doses) and iii) at days 5^th^, 7^th^, 9^th^ 11^th^ (i.e., 4 total injected doses). One-dose injection had been previously shown to be not active at all, in preliminary studies (not shown). According to the schedule followed, treatment was carried out when metastases were already formed and growing. On day 14^th^ mice were sacrificed and superficial macroscopic lung-metastases were then counted. RAM markedly reduced the number of superficial metastatic foci in a dose-dependent way reaching a significant 70%±15% inhibition (p<0.05 by ANOVA followed by Dunnett's test) at 4 injections schedule. On the contrary, RGDS showed a stable effect around 50% inhibition, not statistically significant (p>0.05 by ANOVA followed by Dunnett's test) (Figure 7C).

## Discussion

The RGD *motif* occurs in several ECM proteins and is involved in integrin-mediated cell adhesion, cell survival, invasion, blood coagulation. RGD-containing peptides are currently used in tumor imaging, cell-targeting and radio-treatment [2], [10], [19], [33]. We and Others demonstrated that RGD peptides, besides the extra-cellular anti adhesive effect, may internalize into different cell-types, including melanoma and endothelial cells, and recognize intracellular targets involved in cell-survival [23], [24], [25], [26], [34]. According to these findings it is possible to hypothesize, besides the well recognized extracellular anti adhesive activity, an additional intracellular role of RGD-containing peptides released from the extracellular matrix during physiologic and pathologic tissue-remodeling [35]. Such considerations foster renewed interest on novel clinical use in pathological conditions. In fact many studies aim at developing novel RGD non-peptide analogs with increased efficacy and resistance to proteolytic degradation. RGD peptides show a short half life and are quickly metabolized *in vivo*; for this reason they require high doses to reach acceptable anti metastatic effects *in vivo* [13], while low (namely, nanomolar) concentrations are reported to paradoxically stimulate tumor growth and tumor angiogenesis, under certain experimental conditions [36]. RGD containing peptides showing duplication and circularization [17], [18], [31], or non-peptidic analogs with higher activity and/or higher stability *in vivo* [19], [20], [21], [22] may overcome such limitation, at least in part. We previously demonstrated that both RGDS and its novel analog RAM exert biological effects likely un-related to their anti adhesion activity; in fact they both show strong biological effects onto collagen IV seeded endothelial cells [23], [27]. Collagen IV is known to mediate cells adhesion requiring mostly RGDS-independent integrins [1]. While both RGDS and RAM strongly inhibit cell-adhesion to plastic and vitronectin as well as angiogenesis *in vivo*, on the other hand they do not significantly inhibit endothelial cell adhesion onto collagen IV, and have clear pro apoptotic effects onto collagen IV–seeded HUVEC. Therefore we concluded that RGDS and RAM have biological effects onto endothelial cells dependent and independent from their anti adhesion activity. A clear anti-melanoma activity of RGDS has been recently observed, by direct targeting intracellular molecules involved in the apoptotic cascade, further confirming that RGDS acts via novel mechanisms, additionally to the known anti adhesive effects [24]. In the present study we investigated whether RAM mimics at least in part the effects of RGDS on human metastatic melanoma cells *in vitro* as well as *in vivo*. Figures 1 to [Fig pone-0025352-g002] [Fig pone-0025352-g003] 4 of the present study strongly support the conclusion that RAM is a good functional RGDS-analog under several experimental conditions. They were tested in adhesion assays onto several surfaces (namely plastic, vitronectin, fibronectin, von Willebrand Factor, laminin and collagen IV showing similar anti adhesion effects onto surfaces requiring RGD-dependent adhesion, thus indicating similar specificity for RGD-related integrins. However not exactly overlapping effects were observed in the assay involving laminin-integrin receptors (namely α_3_, α_6_, α_7_ integrins) suggesting a different specificity for these receptors. Several data reported here indicating common and different effects of RGDS and RAM support the hypothesis that the two molecules may have common targets and in part different targets.

Most interestingly, when FCS-induced degradation was allowed, RAM appeared to be significantly more potent that RGDS, both in plastic-seed and collagen-IV- seed conditions (Figures 5, 6). The cyclo-RGDS peptide, i.e. a RGDS-analog developed as a potent anti adhesive molecule [31], was completely inactive under collagen IV- seed conditions (Figure 6), further confirming that RAM exerts biological effects unrelated to the anti adhesion properties, in the presence of serum. Furthermore, such evidence allowed us to conclude that RAM maintains its effect in the presence of serum, while RGDS almost completely loses it upon FCS pre-incubation (Figure 5A–B). RGDS degradation in the presence of serum is a known phenomenon, and synthesis of non-peptidic analogs such as RAM is specifically intended to overcome this limitation and to allow further *in vivo* investigation, in tumor and non-tumor animal models. The present study supports the hypothesis that serum components, likely proteases, may degrade RGDS, abolishing its potent anti-mitogenic effect evident *in vitro* in the absence of serum, while they do not affect RAM activity. While RAM appears to be more potent than RGDS in the presence of serum (Figure 5 and Figure 6), the two molecules show closer efficacy in the presence of FGF-2 *in vitro* (Figure 4), i.e. in the absence of serum-induced degradation. Therefore Figures 1 to [Fig pone-0025352-g002] [Fig pone-0025352-g003] [Fig pone-0025352-g004] [Fig pone-0025352-g005] 6 and Figure S2 suggest that RAM has higher stability and at least partially different target specificity.

All the above considerations suggest that RAM may be more potent *in vivo* as compared to RGDS, indicating RAM as a good candidate for *in vivo* applications. A pure anti adhesive action may be not sufficient to induce regression of metastases already formed; therefore RAM, due to its serum-degradation resistance and to adhesion-unrelated properties, was hypothesized to induce metastasis-regression and to show clear anti-tumor activity in an *in vivo* model. In a mouse melanoma-lung metastasis model RGDS and RAM were therefore injected intravenously at similar molar concentration and increasing doses. RAM strongly and significantly reduced in a dose dependent way the number of lung superficial metastases as compared to controls, while RGDS anti-metastatic effect was less potent and dose-independent (Figure 7). These results suggested that RAM and RGDS *in vivo* anti-melanoma action may be due to a combined anti adhesive and non-anti adhesive mechanism and that RAM may have higher potency due to the higher resistance to FCS-induced degradation. Other Authors previously tested the effect of RGD-analogs in mouse melanoma models [37], [38], [39]. In these studies (differently from the present study), melanoma cells were co-injected intravenously with the RGD-analogs, showing a marked inhibition of lung metastasis achieved by an anti adhesive mechanism, acting onto the adhesion of metastatic cells to the lung tissue. In one other study the potent effect was totally lost by injecting the RGDS analog onto already formed metastases [4]. This study indicated once again that pure anti adhesive molecules may show a potent anti-proliferation effect but may lack activity onto metastases already formed. Differently from these previous studies, in the present study treatment was started at least 5 days after cell inoculation, i.e. when cells are already adhered and metastases are growing already. Under such conditions RAM exerted a dose-dependent and significantly more potent effect than RGDS. Experimental conditions followed in the present study may therefore better re-capitulate clinical conditions and the collected data likely indicate novel relevance for possible clinical applications.

According to a report we published recently [24], RGDS may induce melanoma apoptosis by directly recognizing intracellular targets such as pro-caspases and survivin. Figure 4A shows that RAM and RGDS have similar intracellular effects on pro-caspase-3 expression and survivin expression. Although further studies are needed, we hypothesize that RAM may achieve such effects at least in part by an intracellular activity to be further investigated with liposome-based formulations aimed at facilitating the intracellular targeting.

While further histological and toxicological studies are needed, the present study represents the first demonstration, at our knowledge, that a RGDS-analog shows marked anti-metastatic activity in an *in vivo* model of growing melanoma metastases.

## Materials and Methods

### Ethics statement

The present study has been carried out in compliance with the Italian National Direction n. 86/609/CEE, which regulates animal-care procedures in *in vivo* experimentations. Experimental procedures were performed within the protocol deposited according to Decreto Legislativo 116/92 at the review board of Università Cattolica del Sacro Cuore, Roma, approved with the identification number A39B.

### Peptide synthesis

RGDS peptide (Arginine-Glycine-Aspartic acid-Serine) and Cyclo(-Arg-Gly-Asp-D-Phe-Val) (Cyclo-RGDS) were purchased from Bachem (Bubendorf, Switzerland). RGDS-analogue named RAM was designed to lack peptide bonds, as previously reported in detail [27], [30] and was synthesized by NeoMPS SA (Strasbourg, France) with a purity >95%. RAM structure is H_2_N-Arg-D-Phe-Arg-Malonyl-Asp-NH_2_ and is reported in Figure S1. Two separate preparations have been used throughout this study, showing similar results.

### Cell culture

Human metastatic melanoma cells line SK-MEL-110 were obtained from Gorospe et al. [40]; mouse lung metastatic B16F10 melanoma cells and human malignant melanoma SK-MEL-28 were from ATCC (Manassas, VA). SK-MEL-110 cells showed the expected microscopic phenotype and grew as expected in the presence and in the absence of serum or growth factors. Data obtained on SK-MEL-110 were validated *in vitro* and *in vivo* on B16F10 mouse melanoma cells and *in vitro* on SK-MEL-28 human melanoma cells, both authenticated by ATCC (not shown). Cells were grown as previously reported [41] in DMEM (Hyclone, Logan, UT) supplemented with 2 mM L-glutamine, 100 IU/ml penicillin-streptomycin (Gibco, Invitrogen corporation, Carlsbad, CA), and 10% heat-inactivated FCS (Hyclone, Logan, UT), at 37°C in a 5% CO_2_ atmosphere.

### Cell adhesion assay

Cell adhesion to ECM glycoproteins or to plastic was quantified as previously reported [23]. Briefly, SK-MEL-110 cells were suspended in DMEM plus fresh FCS 10% with serial dilutions of RGDS or RAM ranging from 0 to 500 µg/ml. In molecular aging experiments, molecules were pre-incubated for different time points (0.5, 1, 24, 48 hours) at 37°C in 100% fetal calf serum before adhesion assay, to test loss of activity. Cells were pre-incubated with the “*aged*” molecules at 37°C for 15 minutes and then seeded (15000 cells per well) at 37°C for one hour in 96 well plates pre-coated overnight at 4°C with vitronectin, or laminin, or fibronectin, or von Willebrand Factor or collagen IV (Becton Dickinson, Bradford, MA) (50 µg/ml diluted in PBS, pH 7.4; fibronectin 10 µg/ml diluted in PBS, pH 7.4). Adhesion onto plastic was also tested. Non-adherent cells were discarded by repeated washes, then adherent cells were fixed with 4% formaldehyde in PBS, pH 7.4, for 10 minutes at RT and stained with 0.5% toluidine blue (Merck KgaA, Darmstadt, Germany) in 4% formaldehyde for 10 minutes at RT. Plates were then rinsed extensively with water and stain was extracted by incubation with sodium dodecyl sulfate (SDS) 1% in PBS, pH 7.4, for 30 minutes at RT. Cell adhesion was then quantified as optical density (OD) at 595 nm.

### Proliferation assay, cell cycle analysis and apoptosis

SK-MEL-110 human melanoma cells were assayed as previously reported [24]. They were plated in 6 well plates (8×10^4^ cells/well) onto plastic or onto collagen IV (50 µg/ml) and were allowed to grow for 24 h in DMEM 10% FCS at 37°C. Medium was then replaced with DMEM serum-free for 24 h. Subsequently, cells were exposed to RGDS or RAM dissolved in complete medium or in DMEM containing FGF-2 (Pierce Endogen, Rockford, USA), for 24 h or 48 h at 37°C. Then, cells were photographed, harvested by trypsin-EDTA and counted using hemacytometer. All experiments were carried out at least 3 times in duplicate.

Additional proliferation experiments were carried out with the specific aim to test RGDS, RAM and cyclo-RGDS stability in the presence of serum; SK-MEL-110 cells were exposed for 24 h to RGDS or RAM or cyclo-RGDS (500 µg/ml) pre-incubated for 24 h at 37°C in 100% fetal calf serum (FCS).

The effect of RAM and RGDS on DNA synthesis was performed by combination of bromodeoxyuridine (BrdU) and propidium iodide staining. Treated SK-MEL-110 were incubated for the last 30 minutes with 20 µmol/L BrdU (Sigma) and then fixed with 70% ethanol, according to a previously reported procedure [42].

In cell cycle studies, SK-MEL-110 human melanoma cells were plated in 6 well plates (8×10^4^ cells/well) onto collagen IV (50 µg/ml) and were allowed to grow for 24 h in DMEM 10% FCS at 37°C. To synchronize SK-MEL-110, exponentially growing cells were treated with DMEM 10% FCS containing 100 ng/ml nocodazole (Sigma), for 16 h and then released, as needed, into drug-free medium. The nocodazole mother solution was dissolved in dimethyl sulfoxide (DMSO) stored at −20°C. Control cultures received an equivalent amount of DMSO. After 16 h medium was replaced with fresh DMEM 10% FCS containing RGDS or RAM and cell were treated for 6 h and 24 h. Cells then were harvested by trypsin-EDTA, fixed in ice-cold 70% ethanol and stained with propidium iodide at final concentration of 10 µg/ml [24]. Cell cycle analysis was performed by propidium iodide staining using a FACSCalibur, Becton Dickinson flow cytometer and Cell Quest software for quantification of PI-positive cells [42]. FACS measurements were performed on three independent synchronization experiments.

Apoptosis was assayed on cells seeded onto collagen IV, by analyzing sub-G1 phase and by TUNEL assay (terminal deoxynucleotidyl transferase-mediated dUTP nick end-labeling) and nuclear staining with the DNA-binding fluorochrome Hoechst 33258 (1 µg/ml) (Sigma) [23]. Nuclei were examined with an Axioplan 2 microscope (Zeiss). The number of apoptotic nuclei was determined by counting 10 different fields (400× magnification) per sample.

### Cell Invasion

FCS 10%–induced melanoma cells invasion was measured in modified Boyden chambers as previously reported [23]. Briefly, 8-µm pore-size polycarbonate filters (Costar, Cambridge, MA) were coated with murine collagen type IV (10 µg/ml) for one hour. Growing SK-MEL-110 were harvested by trypsinization, re-suspended in DMEM 0.1% BSA, and 200 µL was added to the upper portion of the chambers at 1×10^6^ cells/ml in the presence of RGDS or RAM (500 µg/ml); the lower portion of the Boyden chamber contained complete medium as chemoattractant. After 4 hours at 37°C, cells were fixed in 95% ethanol and stained with Giemsa (Merck KGaA, Darmstadt, Germany) for 10 minutes. The number of migrated cells was evaluated by counting 15 fields at ×400 magnification.

### Western blotting

SK-MEL-110 seeded on collagen IV and treated for 48 h with FGF-2 in the presence of RGDS or RAM (500 µg/ml), were lysed with RIPA buffer [23]. Samples were boiled, loaded and separated by SDS-PAGE and transferred to nitrocellulose membrane. Membrane was blocked with 5% milk (Bio-Rad Laboratories) in TPBS (0.1% Tween 20 in PBS, pH 7.4), washed and incubated with mouse anti-survivin (1∶200) (Santa Cruz Biotechnology, Santa Cruz, CA), rabbit anti-caspase 3 (1∶200) (Santa Cruz Biotechnology, Alexa, CA,), or mouse anti-β-actin (1∶5000) (Sigma-Aldrich, St Louis, MO) in milk 5% TPBS 0.1% for 1 h at RT. Horseradish peroxidase-conjugated secondary antibodies (Pierce) were used, followed by chemiluminescence assay (ECL; Amersham, Buckinghamshire, United Kingdom) and autoradiography.

### *In vivo* experiments for lung metastasis

*In vivo* experiments for lung metastasis were performed according to an accepted animal-study protocol. Three months-old male C57BL6/J mice (12 mice per group) (Charles River, Wilmington, MA) received an i.v. injection of B16F10 cells (2×10^5^/200 µl in PBS) into the tail vein for experimental metastasis studies at day 0 [43]. Mice were then treated according to a modified schedule derived from the literature [32] with some modification, receiving repeated i.v. injections of 200 µl of identical molar doses of RGDS or RAM (2.6 mM in PBS) (corresponding to 1.25 mg/ml/dose RGDS and 1.8 mg/ml/dose RAM, corresponding to 8 mg/kg/dose and 12 mg/Kg/dose, respectively). RGDS and RAM treatments were carried out according to three different schedules: i) injection at days 9^th^ and 11^th^ (2 total injected doses), ii) injection at days 7^th^, 9^th^ and 11^th^ (3 total injected doses) and iii) injection at days 5^th^, 7^th^, 9^th^ 11^th^ (4 total injected doses These schedules were chosen to start treating mice 5, 7 and 9 days after cell inoculations respectively, i.e. when lung metastases are already developing, in order to study metastases-regression, rather than cell-adhesion to the lung tissue.

Mice were sacrificed at day 14^th^ and lung-superficial macroscopic metastases were counted using a magnifying glass (20×).

### Statistical analysis

All *in vitro* and *in vivo* efficacy experiments were performed at least three times in duplicates. Student's t test was carried out in most cases. To analyze *in vivo* effects, one-way ANOVA test followed by Dunnett's Multiple Comparison Test as post-hoc analysis were carried out with PRISM software; p<0.05 was considered the statistically significant threshold.

## Supporting Information

Figure S1 **RAM molecular structure.**
(TIF)

Figure S2 **The** ***in vitro*** **anti proliferation effect of RGDS and RAM (500 µg/ml) on collagen IV was investigated in the presence of FCS in SK-MEL-28 human melanoma cell line.** RAM shows a significant anti proliferative action, while RGDS is inactive. Onto SK-MEL-110 human melanoma cell line RGDS has a weak effect while RAM has strong inhibitory effect (see Fig. 2D).
(TIF)
